# The effect of earplugs during the night on the onset of delirium and sleep perception: a randomized controlled trial in intensive care patients

**DOI:** 10.1186/cc11330

**Published:** 2012-05-04

**Authors:** Bart Van Rompaey, Monique M Elseviers, Wim Van Drom, Veronique Fromont, Philippe G Jorens

**Affiliations:** 1 University of Antwerp, Faculty of Medicine and Health Sciences, Universiteitsplein 1, 2610 Wilrijk, Belgium; 2 Artesis University College of Antwerp, Department of Health Sciences J. De Boeckstraat 10, 2170 Merksem, Belgium; 3 University of Antwerp, Faculty of Medicine and Health Sciences, Division of Nursing Science and Midwifery; Universiteitsplein 1, 2610 Wilrijk, Belgium; 4 Antwerp University Hospital, Intensive Care Department, Belgium

## Abstract

**Introduction:**

This study hypothesised that a reduction of sound during the night using earplugs could be beneficial in the prevention of intensive care delirium. Two research questions were formulated. First, does the use of earplugs during the night reduce the onset of delirium or confusion in the ICU? Second, does the use of earplugs during the night improve the quality of sleep in the ICU?

**Methods:**

A randomized clinical trial included adult intensive care patients in an intervention group of 69 patients sleeping with earplugs during the night and a control group of 67 patients sleeping without earplugs during the night. The researchers were blinded during data collection. Assignment was performed by an independent nurse researcher using a computer program. Eligible patients had an expected length of stay in the ICU of more than 24 hours, were Dutch- or English-speaking and scored a minimum Glasgow Coma Scale of 10. Delirium was assessed using the validated NEECHAM scale, sleep perception was reported by the patient in response to five questions.

**Results:**

The use of earplugs during the night lowered the incidence of confusion in the studied intensive care patients. A vast improvement was shown by a Hazard Ratio of 0.47 (95% confidence interval (CI) 0.27 to 0.82). Also, patients sleeping with earplugs developed confusion later than the patients sleeping without earplugs. After the first night in the ICU, patients sleeping with earplugs reported a better sleep perception.

**Conclusions:**

Earplugs may be a useful instrument in the prevention of confusion or delirium. The beneficial effects seem to be strongest within 48 hours after admission. The relation between sleep, sound and delirium, however, needs further research.

**Trial registration:**

Current Controlled Trials ISRCTN36198138

## Introduction

Delirium is a common complication in the ICU caused by a malfunction of the cognitive processes in the brain. The syndrome is characterized by a fluctuating course, shifting attention, disorganized thinking and a changed level of consciousness [1]. Incidences from 20% to more than 80% are reported in different patient groups using different assessment tools. Predisposing and precipitating risk factors related to patient characteristics, chronic pathology, acute illness and the environment have been studied [2,3]. A patient encountering three or more of these factors has a 60% increased risk for the development of delirium [2,4]. Ely *et al. *stated that a patient in the ICU even accumulates ten or more of these factors [5].

Delirium often presents early after admittance to the ICU. The early onset is probably caused by an acute change in the physical situation of the patient stressed by a sensory overload. A few days after admission a cognitive healthy patient may shift to a delirium due to underlying biomedical changes or worsening illness. In this context, delirium may be called the sixth vital sign [6,7]. Next to delirium, confusion is mentioned as a symptom in different psychiatric and cognitive disorders and is described as 'a state of disturbed orientation in regard to time, place or person, affecting the clarity and the coherence of one's thinking' [8]. Consequently, patients classified as confused have an altered perception or thought but may score negative for delirium.

The ICU is a rapidly changing ward designed to admit severely ill patients. The typical character and the health care process in this unit induce heavier care sustained by high technological equipment. This equipment and the higher intensity of care also produce augmented sound levels [9]. Sound in the ICU has been a subject of research for years. Peak noise is not the main determinant disturbing the patient in the ICU. Phones ringing and people talking are reported as more annoying [10]. Although often suggested, there is ample evidence that sound influences the patient's outcome. Most studies on noise report on a possible relation with sleep or on results of architectural improvements [11-14]. The quality of sleep in the ICU, however, has been related to environmental sound [10-12,15,16]. Moreover, the impact of disturbed sleep on the onset of delirium in the ICU has been proposed. Several studies showed severe fragmentation, arousals and awakenings in the sleep of ICU patients and pointed at the absence of slow wave and REM sleep. Researchers hypothesized that this disturbance of sleep could be an important role player in the onset of the delirious syndrome [17,18].

Although the impact of sleep on the onset of delirium has often been suggested, sound influencing sleep has not been identified as a risk factor for delirium yet. We hypothesized that a reduction of sound during the night using earplugs could be beneficial in the prevention of the early onset of intensive care delirium. Two primary research questions were formulated. First, does the use of earplugs during the night reduce the onset of delirium in the ICU? Second, does the use of earplugs during the night improve the quality of sleep in the ICU?

## Methods

This study was a randomized clinical trial in which adult intensive care patients were assigned in a 1:1 ratio to an intervention group, patients sleeping with earplugs during the night, or a control group, patients sleeping without earplugs during the night. The researchers were blinded during data collection. Assignment to the study or control group was done by an independent nurse researcher using a computer program. Since the focus of this study was the early onset of delirium, patients were to be observed during a maximum of five nights. Earlier research in the same setting showed that most delirium cases presented in the first 72 hours after admission to the ICU [3,19,20]. Patients scoring positive for delirium were censored for further observation and analysis.

### Participants and study settings

All patients were admitted to the intensive care department of the Antwerp University Hospital (625 beds). The department has a capacity of 45 beds admitting more than 2,600 patients each year. This department is divided into different units (7 to 15 beds each). These units are preferentially but not exclusively specialized in treating cardiac-surgical, surgical or medical ICU patients. Patients are admitted to a separated space or an individual room, each with a clock, visual and auditive contact with the staff and the possibility to listen to the radio or watch television. Most of the patients (> 75%) have a window with visible daylight.

Eligible patients were all adults (18 years old or older). They were included when the expected length of stay in the ICU was more than 24 hours, when speaking Dutch or English and scoring a minimum Glasgow Coma Scale of 10. Patients with known hearing impairment, dementia, confusion or delirium at admission were excluded. Also, sedation was used as an exclusion criterion to optimize the assessment of delirium and sleep perception. Data collection took place from 21 November 2008 until 1 April 2009 and from 1 November 2009 until 1 April 2010. This collection included baseline patient data, Richmond Agitation and Sedation Scale score (RASS), and Glasgow Coma Scale and Neelon and Champagne Confusion Scale (NEECHAM) as validated scoring systems for agitation, delirium and consciousness score [19]. The study and the control groups were compared for severity of disease using the Simplified Acute Physiology Score 3 (SAPS 3) score [21], for organ failure and dysfunction using the Sepsis-related Organ Failure Assessment (SOFA) score from the first 24 hours [22] and for acute kidney injury using the maximum Risk, Injury, Failure, Loss and End Stage (RIFLE) score during the study [23]. The patients requiring sustained low-efficiency daily dialysis (SLEDD) were also counted in each group [24]. Nursing activity was compared for both groups using the Simplified Therapeutic Intervention Scoring system (TISS 28) [25]. Based on the experience of the research group standardized forms were used to observe environmental and other known risk factors for delirium [3]. Additional data for the included patients on ventilation and patient characteristics are presented.

The sample size was calculated based on our earlier findings [3]. The incidence of delirium was 29.6% and mild confusion was 25.8%. We hypothesized that the use of earplugs could lower the incidence of delirium or confusion by 20%. Sample size calculation with a power of 0.80 and α = 0.05 showed that 46 patients had to be included in the study group and the control group.

### Intervention and randomization

All intensive care nurses and physicians were informed before starting the study. A poster summarizing the study protocol was visible at all times on every unit. The researchers screened all intensive care patients on a daily basis to invite eligible patients to the study. After giving informed consent, an independent nurse researcher assigned the patients to the study group or the control group using a list generated by a computer program. Next, a nontransparent canister holding earplugs or a dummy was positioned at the bedside of the patient.

The researchers activated a reminder in the electronic patient data record system (iMD Soft, Metavision). This reminder assigned the critical care nurse at 22.00 hours (start of the night shift) to open the non-transparent canister and to position the earplug when present. A second assignment at 06.00 hours (before the end of the night shift) asked the critical care nurse to remove the earplugs from the patient and to keep them in the closed canister again. When the canister contained a dummy instead of earplugs, no action was undertaken. Patients and staff were instructed not to report on wearing or not wearing earplugs during the night to the researchers. One of the blinded researchers visited the patients during the morning to assess them for delirium and sleep perception.

The study group selected the polyurethane Bilsom type 303 SNR 33 dB(A) earplug (Howard Leigh Honeywell, San Diego, CA, USA). This commonly used earplug is cheap, easy to use and had a guaranteed delivery during the study. The selected device lowers the perception of the environmental sound by 33 decibels [26].

### Assessment of delirium and confusion

The primary outcome of this study was to lower the prevalence of delirium in the study group compared to the control group. Delirium was assessed using the NEECHAM. Earlier research showed this tool, after being translated into Flemish [27], to be valid in an ICU population [19,28]. Moreover, the nurses and the research staff on the ICUs were already used to assessing the patients for delirium with this tool. No additional training of the research team or critical care nurses was required.

The NEECHAM is based on the nurses' twenty-four hour assessment of the level of processing information, the level of behavior and the physiological condition, rating the patient on a 30 to 0 scale. Next, the results can be classified in one of four categories. The cut-off values, 30 to 7 'normal', 26 to 25 'at risk', 24 to 20 'early to mild confusion' (mild confusion) were standardized. The scores 19 to 0 'moderate to severe confusion' indicate delirium in the studied patient. The NEECHAM was assessed each nursing shift, at 08.00 hours, 16.00 hours and 22.00 hours. The nurse taking care of the patient during the evening shift scored the second and the third NEECHAM. The night shift nurse applied and removed the earplugs. Consequently, the research nurse and the critical care nurse scoring the NEECHAM had no information on the use of earplugs

### Assessment of sleep perception

The second primary outcome in this study was sleep perception in intensive care patients using or not using earplugs. Sleep perception was assessed using five dichotomous questions on the self-reported sleep quality of the patient: 1) Did you sleep well? 2) Did you sleep better than expected? 3) Did you sleep better than at home? 4) Were you awake for a long time before falling asleep? 5) Do you feel sufficiently rested? The score on question four was reversed. A higher total sum score on the five questions showed a better sleep perception. The scores were categorized as bad sleep (sum < 2), moderate sleep (2 ≤ sum < 4) and good sleep (4 ≤ sum).

### Statistical methods

All data were analyzed using the Statistical Package for the Social Sciences 16.0 (SPSS). Differences between the study and the control populations were calculated using the Student's t-test, Mann-Whitney U and the Pearson's Chi-square where appropriate. The level of significance was 0.05 for all tests.

The patient's lowest score for the NEECHAM during the study was registered for the calculation of the incidence of delirium or mild confusion. The NEECHAM scale was handled as a semi-quantitative scoring system. Therefore, differences between the study and the control groups were calculated using non parametric statistics.

Survival life table analysis was used to study the outcome 'delirium or mild confusion' in both groups. Significance was calculated using Wilcoxon log rank. Multivariate analysis using 'delirium or mild confusion' as dependent outcome variable was done with Cox regression. Patient characteristics and studied risk factors for delirium were stepwise forward added to the model. The probability for stepwise was set at entry level 0.05 and removal at 0.10. Hazard ratios were calculated with a 95% CI.

### Ethical considerations

The study was approved by the ethical board of the Antwerp University Hospital in November 2008 with reference number 8/40/223. The trial was registered in the Current Controlled Trials database (ISRCTN36198138). Each participant gave informed consent to the study and was individually informed by a researcher. All data were anonymized. The study did not interfere with daily care or treatment of any of the patients. When a patient's condition or illness worsened too much within the first 24 hours, the patient was excluded from further participation. This study was not funded nor were there any relations or contacts with the supplier of the earplugs.

## Results

**Figure 1:**
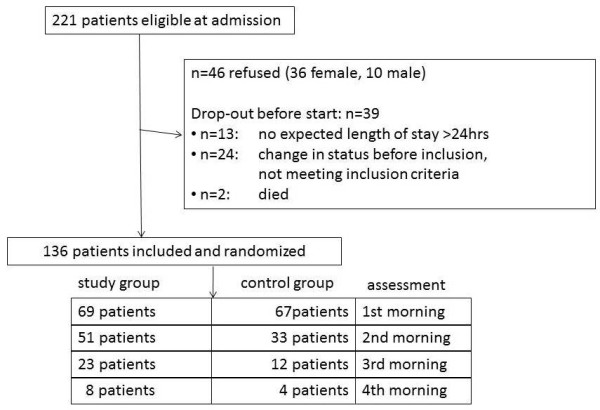
**Flowchart showing selection and inclusion of patients**.

During the study period 221 patients were found to be eligible in four ICU-subdivisions. After being informed, 46 patients, 36 women and 10 men, refused further cooperation. An early drop out before the randomization was caused by an unexpected length of stay of less than 24 hours (*n *= 13) or severe worsening of the patient's condition or illness (*n *= 24). Consequently, these patients, not meeting the inclusion criteria, were not randomized. Additionally, two patients died before the first assessment of delirium and sleep perception. The study population comprised 136 patients, 69 in the study group and 67 in the control group (Figure 1). The mean age of the population was 59 years (range18 to 84), 66% were men. The mean SAPS 3 was 42.3 (0 to 78), the mean SOFA score during the first 24 hours was 7.1 (1 to 14) and the mean TISS 28 score was 24.5 (9 to 43). The patients using earplugs had a significantly longer observation period than the control group (43 hours versus. 33 hours, *P *= 0.02). During the maximum observation of five days, 20% of the patients were delirious and 27% showed mild confusion on at least one observation moment. Additionally, the NEECHAM assessments showed 23% of the patients were at risk for delirium and 30% were classified as normal. Most included patients stayed only one night in the ICU. Both study groups were comparable at baseline as few statistical differences were found between the study and the control groups (Table 1).

**Table 1:** Description of population and relevant risk factors for delirium.

			Study Group *N *= 69	Control Group *N *= 67	*P*-value
Patient	age	years, mean (range)	57 (19 to 81)	62 (18 to 84)	0.57
characteristics	gender	male	68.1%	64.2%	0.72
	education	university	7.2%	11.9%	0.07
		college	40.6%	19.4%	
		high school	42.0%	55.2%	
		other	10.1%	13.4%	
	smoking	daily smoking	20.3%	23.9%	0.68
		number of cigarettes per day when smoking	17.4	11.8	0.14
	alcohol	regular use	46.4%	40.3%	0.49
		number of units/week when regularly use	6.7	5.8	0.65
	living single at home	yes	18.8%	28.4%	
	professionally active	yes	46.4%	31.3%	0.01
	kids	yes	73.9%	73.1	0.92
Chronic pathology	≥ 1 Comorbidity	yes	68.1%	75.8%	0.32
Acute illness	TISS 28	mean (range)	24.5 (9 to 40)	24.5 (11 to 43)	0.74
	SOFA score first 24 hrs	mean (range)	7.2 (1 to 14)	7.0 (2 to 15)	0.65
	SAPS 3 score	mean (range)	42.5 (0 to 78)	42.1 (0 to 78)	0.89
	admission	surgery	69.6%	79.1%	0.20
		internal medicine	30.4%	20.9%	
		emergency surgery versus scheduled research	21.6%	29.7%	0.30
	first time intensive care	yes	65.2%	44.8%	0.02
	Maximal RIFLE	No acute kidney injury	3.1%	9.5%	0.22
	score during study	risk	9.2%	3.2%	
		injury	20.0%	15.9%	
		failure	67.7%	71.4%	
	SLEDD necessity	Number of patients	4	5	0.52
	length in study	mean hours of observation per patient (SD)	42.8 (25.7)	32.6 (25.7)	0.02
Environment	intensive care unit	study unit 1	21.7%	17.9%	0.57
		study unit 2	21.7%	26.9%	
		study unit 3	29.0%	20.9, %	
		study unit 4	27.5%	34.3%	
	visible clock	yes	95.7%	89.6%	0.17
	visible daylight	yes	60.9%	67.2%	0.45
	isolation	yes	2.9%	6.0%	0.38
	no visit	yes	0.0%	4.5%	0.12
	room	open	39.1%	56.7%	0.12
		separated by walls, open end	18.8%	11.9%	
		closed box	42.0%	31.3%	

*P*-value was calculated using independent-samples T test, Mann Whitney U test or Chi square where appropriate. N, number; RIFLE, Risk, Injury, Failure, Loss and End Stage; SAPS 3, Simplified Acute Physiology Score 3; SLEDD, sustained low-efficiency daily dialysis; SOFA, Sepsis-related Organ Failure Assessment; TISS 28, Therapeutic Intervention Scoring System.

### Delirium and earplugs

**Figure 2:**
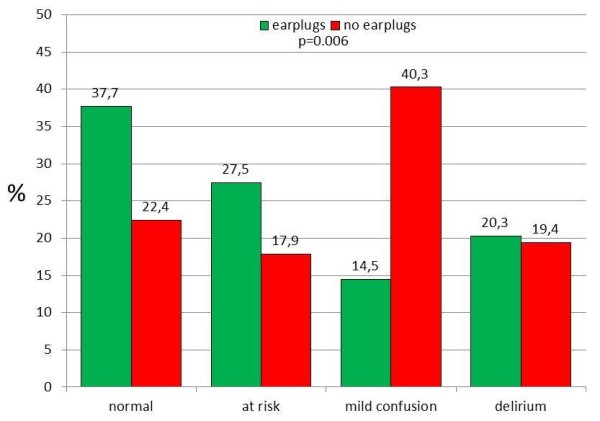
**NEECHAM categories observed for the study (earplugs) and the control group (no earplugs) using the worst score during the observation period of maximum five nights**. Chi^2 ^for difference between earplugs and no earplugs: *P *= 0.006.

The study group, sleeping with earplugs, showed a median NEECHAM score of 26 (5 to 29) and the control group 24 (8 to 29) (Mann-Whitney U, *P *= 0.04). More cognitively normal patients were found in the group sleeping with earplugs (*P *= 0.006) (Figure 2). The study group scored 19% delirium, the control group 20%. The major difference was observed in the mild confusion group. Patients sleeping with earplugs showed 15% mild confusion, whereas the control patients scored 40% in this category. Taking both categories, delirium and mild confusion, into account, 60% of the control group showed cognitive disturbances against only 35% in the study group.

**Figure 3:**
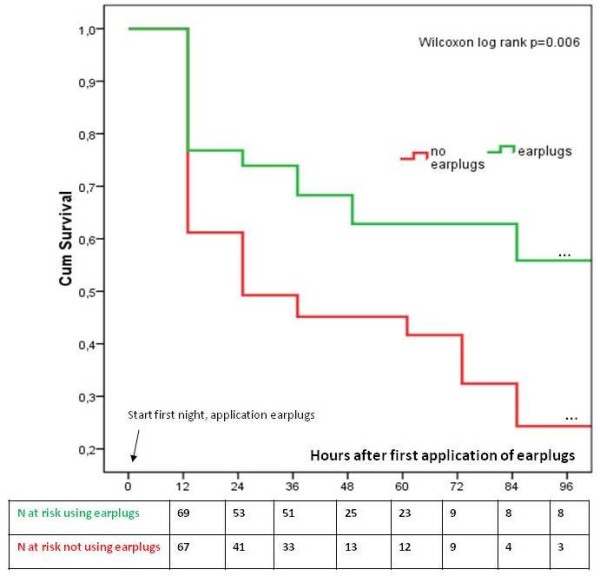
**Life-time table analysis: time until first delirium or mild confusion (NEECHAM ≤ 24) for the study (earplugs) and the control group (no earplugs)**.

Survival analysis showed a strong benefit for the prevention of cognitive disturbances in favor of the earplugs within the first 24 hours. This beneficial effect was sustained during the observation period (Wilcoxon log rank, *P *= 0.006) (Figure 3). Cox regression revealed that the use of earplugs decreased the risk of delirium or confusion by 53% (HR .0.47, CI 0.27 to 0.82). The use of earplugs was corrected for all patient characteristics and risk factors for delirium mentioned in Table 1. In the multivariate model, the risk for delirium or confusion also increased by 3% per year for age (HR 0.47, 95%CI 1.01 to 1.05), by 9% for each increase in points of the SOFA score (HR 1.09, 95%CI 1.01 to 1.17) and by 87% for patients who smoked (HR 1.97, 95%CI 1.10 to 3.51) (Table 2).

**Table 2:** Hazard ratio's for delirium or mild confusion.

Factor	*P*	HR	95%CI for HR
Earplugs	0.008	0.47	0.27-0.82
SOFA (per point increase)	0.024	1.09	1.01-1.17
Age (per year increase)	0.02	1.03	1.01-1.05
Smoking	0.014	1.87	1.10-3.51

The model is significant at *P *= 0.02. The use of earplugs was corrected for all patient characteristics and risk factors mentioned in Table 1. CI, confidence interval; HR, Hazard Ratio; SOFA, Sepsis-related Organ Failure Assessment.

### Sleep perception and earplugs

**Figure 4:**
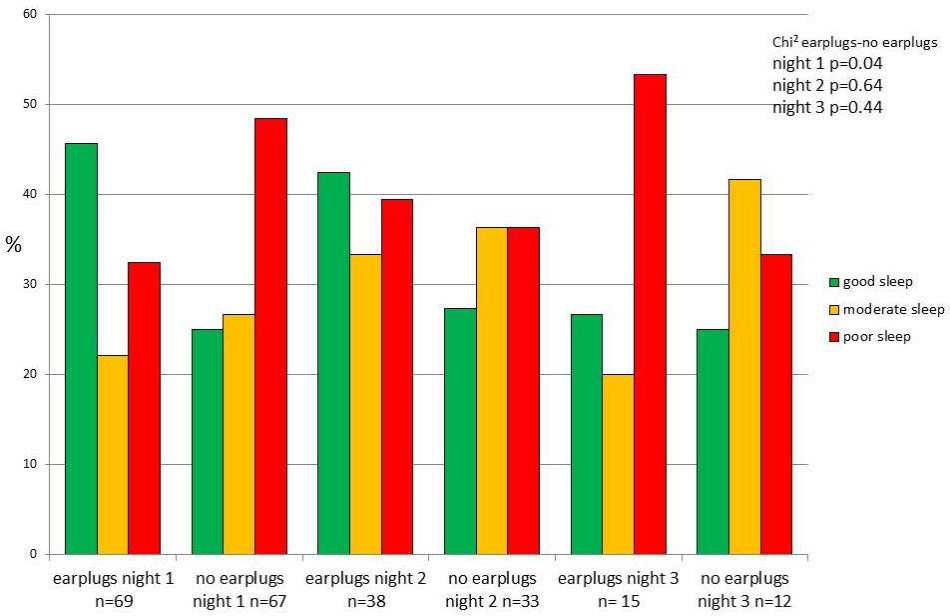
**Sleep perception for the study (earplugs) and the control group (no earplugs)**. Chi^2 ^for difference between earplugs and no earplugs for each night.

The second outcome in this study, the self-reported sleep perception of the patient, was observed in all patients after the first night (Figure 1). Four patients were not able to reply to the questions on sleep perceptions due to an ongoing delirium. Three were in the control group, one patient in the study group. Patients sleeping with earplugs showed a significantly better sleep after the first night (*P *= 0.042). Nearly half of the study group reported a good sleep, whereas only one fourth of the control group reported a good sleep. Almost half of the patients sleeping without earplugs reported a poor sleep after the first night; in the study group one third reported a poor sleep. This significantly beneficial effect was sustained in the second night, although it was no longer significant. After the third night more patients with earplugs reported a poor sleep (Figure 4). After the fourth night too few patients responded to the sleep perception. Therefore, further analysis was not executed.

## Discussion

The use of earplugs during the night proved to be beneficial in our study group. Fewer patients showed delirium or confusion. A vast improvement was shown in the NEECHAM category 'mild confusion'. Moreover, the onset of cognitive disturbances was delayed compared to the patients sleeping without earplugs. Additionally, patients sleeping with earplugs who developed delirium or confusion did not suffer from the syndrome as soon as patients sleeping without earplugs. More patients reported a better sleep perception after sleeping with earplugs in the ICU.

The imbalance of sample size after day one is mainly due to the higher number with 'first signs of delirium' in the control group resulting in the end of observation for these patients. The patients in the control group showed an earlier onset of delirium resulting in a smaller population to study. This resulted in a difference of observation time as presented in Table 1. These findings, however, are not to be considered as a reflection of the actual length of stay in the ICU.

Delirium induced by environmental and sensorial factors appears early during admission to the ICU. A later onset of the syndrome is probably caused by changes in illness, hemodynamic or biomedical situation or ongoing treatment [6,7]. Most researchers describe delirium in the ICU as a multifactorial syndrome [1,3]. Although being the same syndrome, early stage delirium may be different from a 'second period' delirium induced by severity of illness. The focus of our research was on the early onset of the syndrome by observing only the first days after admission of the patient. Known risk factors for delirium were not different for the study and the control groups (Table 1). No differences were observed between the two groups for the known risk factors of delirium.

Since the initial development of the NEECHAM scale, patients are divided into four categories (delirium, mild confusion, at risk and normal). We studied the NEECHAM scale in relation to the CAM-ICU in earlier research. Both scales proved to be comparable in the detection of delirium [19]. Delirium is a syndrome well-defined by the Diagnostic and Statistical Manual of Mental Disorders, Fourth Edition (DSM-IV-TR) criteria [1]. The category mild confusion, however, has been mentioned by several authors without a clear definition [27,28]. The clinical relevance of the NEECHAM category 'confusion' has not been studied thoroughly yet, but may be considered as a prelude to delirium. Hard outcomes for 'mild confusion' have not been studied yet in an intensive care population. More patients remained normal or at risk using the earplugs at night. The category mild confusion was more present in the control group. Consequently, this study pointed to a possible relevance of this category for the first time. Further research needs to focus on the clinical relevance of this category. Meanwhile, it is advisable to observe patients scoring 'confusion' in order to receive focused care attempting to prevent delirium.

The incidence of delirium, however, was not different for both groups. Since it is hard to believe that the exclusion of a single risk factor resolves delirium, the use of earplugs is no magical solution in the prevention of the syndrome. Patients in the study group were triggered by other factors to evoke the confusion or delirium. The multivariate model showed age, smoking and severity of disease to be important role players in this population also. This confirms earlier research [3,5]. The clearly beneficial effect of the use of earplugs, however, is strong enough to advise their use during the night in the ICU. Moreover, patients using earplugs developed delirium or confusion later during their stay in the ICU. A protection in the early stage of the admission to the unit was therefore demonstrated. The effect of mild confusion on the patient's transition into delirium has not been studied yet. Therefore, our interventions must be situated in the prevention of the early onset delirium. Although the harmful outcome of delirium has only been proven for the worst stage, it can also be advised to consider the use of earplugs in the prevention of the early stage of confusion. Mistraletti *et al. *pointed already at the possible profit in the prevention of delirium in improving patient's sleep [18]. This was not proven in our research yet. The beneficial outcome may not be completely studied yet, but it seems obvious that applying earplugs to all patients favors sleep. A larger scale use may be recommended while the outcome of this improved sleep perception can be studied in a larger design.

Poor sleep has been shown in intensive care patients [11,18]. Polysomnography is the golden standard to assess sleep objectively. Since this tool is expensive and very labor intensive, large scale studies are rare and the implementation of polysomnography in a major study to assess sleep seems hard to manage. An objective assessment of sleep, however, is needed in the search for poor sleep as a risk factor for delirium. Self-reported sleep perception as the subjective self-reported assessment of sleep quality is easier to study. Therefore, sleep perception is easier to study as a risk factor but shows some important limitations. Validated scales to assess sleep perception were tested but seemed to create a burden on the intensive care patient because they were too long and required a lot of attention. Therefore, questions were simplified to have an easy response from the patients. Not being validated, the results of the questions must be considered as indicative.

Earlier research showed a development of sound modification programs based on architectonical, structural or staff behavioral interventions. Conversation between staff seems one of the major sources of noise. Therefore, a staff education program could already affect 14% of the peak sound sources [9,29]. Most likely, the architectural structure based on closed rooms reduces most effectively the sound at the bedside as described by Gabor *et al. *[15].

Study patients in this trial reported a better sleep perception due to the noise reduction by earplugs. Hardly any studies have been performed with earplugs in the ICU. On the other hand, some potential beneficial effects providing a reasonable basis for testing the effects of earplugs in critically ill subjects were reported. These cheap devices are capable of reducing the incidence of intraoperative awareness with recall during elective orthopedic surgery [30]. One small randomized study in the neonatal ICU showed a significant effect of silicone earplugs on weight gain in 'very low birth weight' (< 1,500 g) and even better outcomes in 'extremely low birth weight' (< 1,000 g) newborns [31]. Earplugs worn by healthy volunteers during exposure to noise levels as observed in the ICU produce a significant decrease in REM sleep latency and an increase in the percentage of REM sleep [32]. The use of earplugs and eye masks together resulted in improvement of polysomnographic variables such as more REM time, shorter REM latency, less arousal and elevated melatonin levels in a limited group of healthy subjects exposed to recorded ICU noise and light together [33]. The latter study in volunteers, however, explored the effects of both ICU noise and light. We are aware of only one previous study exploring the use of earplugs alone in a real ICU environment. Scotto *et al. *were able to improve the subjective total sleep satisfaction score in non-ventilated, non-sedated adults after the use of earplugs in the ICU but did not explore the effect on delirium [34]. The use of earplugs, however, is cheap, easy and has apparently the same effect on all patients without the necessity to introduce more extensive structural or organizational changes on the ward.

Our study has some limitations. This randomized controlled trial included a specific population in our ICU. Therefore, results may not be applicable to all settings and all patients. Moreover, including this specific population, the findings seem to focus on the first 24 hours of admittance. Larger research may focus on the total length of stay in the ICU of all patients. No accidental or intentional removal of the earplugs was reported. All included patients agreed to sleep with earplugs. Patients who did not like to use earplugs could not give their consent to the study. Including the patients for the trial, the larger group of refusals were women. They indicated that they prefer remaining in direct contact with their environment. Further research could focus on the reasons for this refusal. Also, many patients stayed only one night in the ICU. Consequently, a short term effect of the use of earplugs was studied. A study on the longer term outcome must be included in a larger scale project. Also, the fact of being delirious makes it impossible for patients to report on sleep perception. Other tools must be searched for to study this perception in delirious patients specifically. At that time a validated easy-to-use scale for sleep perception in the ICU may become available.

## Conclusions

Despite the fact that the relationship between sleep perception and delirium has not been clearly established, this study pointed at a relation between environmental sound, sleep perception and delirium. The NEECHAM Confusion Scale showed a significantly lower proportion of patients with mild confusion or delirium in the study group sleeping with earplugs during the night in the ICU. Also, patients reported a better sleep perception using earplugs. Earplugs may be a useful instrument in the prevention of confusion or delirium. The beneficial effects seem to be strongest within 48 hours after admission. The relationship between sleep, sound and delirium, however, needs further research.

## Key messages

• Patients sleeping with earplugs have a 43% lower risk for confusion in the ICU. The beneficial effects seem to be strongest within 48 hours after admission.

• The use of earplugs improves the sleep perception of patients

• Since delirium is a multifactorial syndrome, sleeping with earplugs is no magical solution in the prevention of delirium

• Earplugs are a cheap and easy to use tool to improve the patient's comfort and to prevent confusion.

## Abbreviations

NEECHAM: Neelon and Champagne Confusion Scale; RASS: Richmond Agitation and Sedation Scale; REM sleep: rapid eye movement sleep; RIFLE: Risk, Injury, Failure, Loss, and End stage; SAPS 3: Simplified Acute Physiology Score 3; SLEDD: sustained low-efficiency daily dialysis; SOFA: Sepsis-related Organ Failure Assessment; SPSS: Statistical Package for the Social Sciences; TISS 28: Therapeutic Intervention Scoring System.

## Competing interests

The authors declare that they have no competing interests. This study or the authors were not funded, nor were there any relations or contacts with the supplier of the earplugs.

## Authors' contributions

BVR conceived and coordinated the study which was further designed by BVR and PJ. WVD and VF actively recruited the patients, asked for the informed consent and gathered the data. All authors actively participated during the course of the study. WVD and VF initiated, while BVR and ME fine-tuned the statistical analysis. BVR drafted the manuscript. All authors read and approved the final version of the manuscript.
